# Borderline epithelial tumors of the ovary: Experience of 55 patients

**DOI:** 10.3892/ol.2014.2758

**Published:** 2014-12-02

**Authors:** VERA LOIZZI, LUIGI SELVAGGI, LUCA LEONE, DONATELLA LATORRE, DORIANA SCARDIGNO, FRANCESCAPAOLA MAGAZZINO, GENNARO CORMIO

**Affiliations:** 1 Department of Biomedical Science and Human Oncology, University of Bari, Bari I-70124, Italy; 2 Division of Gynecology Oncology, Scientific Institute for Research, Hospitalization and Health Care Bari, Bari I-70124, Italy

**Keywords:** borderline ovarian tumor, prognostic factors, surgery, follow-up

## Abstract

The objective of the present study was to evaluate the clinicopathological features and the survival time estimates in patients treated for borderline ovarian tumors (BOTs). A retrospective review of all patients treated for BOTs at the University of Bari (Bari, Italy) between 1991 and 2011 was performed. Data were obtained from hospital records and gynecological oncology charts. A total of 55 patients were identified. The median age was 40 years (range, 13–79 years). The majority of the patients (85.5%) exhibited International Federation of Obstetrics and Gynecology (FIGO) stage I disease and the remainder exhibited FIGO stage II/III (7.3% in each stage). Serous histology was found in 60.0% of the cases and an elevation of the cancer antigen-125 serum level occurred in 23.6% of the cases. All patients underwent surgery and 3.7% received chemotherapy. In total, 10.9% exhibited recurrence and the median survival rate was 39 months. The median survival time and the five-year survival rate were 42 months (range, 16–84 months) and 97%, respectively. Therefore, BOTs have an excellent prognosis. Conservative surgery should be considered for patients of reproductive age who desire preservation of fertility. A long-term follow-up is highly recommended for these tumors.

## Introduction

Taylor first described borderline ovarian tumors (BOT) in 1929, characterizing them as ‘semi malignant’ or ‘borderline’ due to the features of these tumors (1). BOTs were later classified by the World Health Organization (WHO) in 1973 as ‘low malignant potential ovarian tumors’ and they were subsequently separated from carcinomas and defined as ‘borderline tumors’ by the WHO in 2003 (2).

BOTs represent 10–20% of epithelial ovarian neoplasms (3). The incidence of BOTs is 1.8–4.8 cases per 100,000 females per year (4), and they typically have an excellent prognosis.

Although BOTs may occur at any age, they are predominantly diagnosed in pre-menopausal females aged 34–40 years old (5).

Unlike invasive carcinomas, BOTs include the key characteristics of cytoplasmic and nuclear atypia, which is an element of differential diagnosis with benign tumors, and an absence of stromal invasion, which is an element of the differential diagnosis with malignant tumors. An unusual degree of proliferation of the epithelial cells with cellular stratification, including notable architectural atypia and the formation of papillary protuberances, is another key characteristic. The absence of evident stromal invasion is a major diagnostic criterion for these tumors (6).

The present retrospective chart review was conducted in order to evaluate the clinicopathological features and the survival time estimates in patients treated for BOTs at the University of Bari (Bari, Italy).

## Patients and methods

The population of the present retrospective study consisted of patients with BOTs who were treated at the University of Bari, referred there for treatment or referred there for follow-up immediately after surgery performed elsewhere during the period between January 1991 and December 2011. All patients with any concurrent invasive malignant disease were excluded from the analysis. Approval was obtained from the Institutional Review Board of the University of Bari for this study and written informed consent was obtained from all patients.

Data, including the age at diagnosis, histological subtype, tumor stage, extent of primary surgery and staging procedures, post-operative treatment and response, date and site of disease recurrence, and current patient status, were collected from hospital charts and gynecological oncology files. A histopathological review of the BOT was performed by the same pathologist for all cases. The staging was based on the International Federation of Obstetrics and Gynecology (FIGO) staging system (7). Histological classification was in accordance with the WHO histological typing of ovarian neoplasms (8). Follow-up procedures included a physical examination and serum cancer antigen (CA)-125 and CA19-9 measurements every three months for two to three years and at 6–12 month intervals thereafter. Additional imaging studies were obtained as clinically indicated based on the above assessment or patient symptomatology.

## Results

### Demographic data

A total of 55 patients with BOTs were identified. The median age at diagnosis was 40 years (range, 13–79 years). The majority of the patients were symptomatic at presentation, usually complaining of abdominal-pelvic pain or pressure. The most common finding upon physical examination was a pelvic mass. The primary tumor diameter ranged between 0.5 and 10 cm, and three (5.5%) of the 55 patients presented with ascites at the time of diagnosis. Of the 55 patients, only 26 (47.3%) underwent a tumor marker evaluation at the time of the initial surgery. Specifically, CA-125 was higher in 13 patients (23.6%), CA 19-9 in two (3.6%) and the two markers were coexpressed in four patients (7.3%). The tumor histology included 33 serous (60.0%), 18 mucinous (32.7%), one endometrioid (1.8%) and three mixed (5.5%) tumors.

### Treatment data

All patients underwent surgery as the primary treatment. In total, 49 patients in the cohort (89.1%) underwent the surgical treatment at the University of Bari. The majority (72.8%) underwent a laparotomic surgery, whereas 13 (23.6%) underwent laparoscopic surgery.

Overall, 20 patients (36.4%) underwent a total abdominal hysterectomy with bilateral salpingo-oophorectomy, whereas only two patients (3.6%) underwent a uterus-sparing bilateral salpingo-oophorectomy and the remaining 33 patients (60.0%) underwent a surgery specifically targeting the adnexa. Omentectomy was performed in 32 patients (58.2%), para-aortic lymph node dissection in only one patient (1.8%) and an appendectomy in 17 patients (30.9%). Peritoneal biopsies were performed in 27 patients (49.1%), whereas peritoneal cytology data was available for 29 cases (52.7%) and was positive in only two of these cases (6.9%). The histological type was serous in 33 patients (60.0%), mucinous in 18 patients (32.7%), mixed in three cases (5.5%) and endometrioid in the remaining one patient (1.8%).

A total of 47 patients exhibited FIGO stage I (85.5%), of whom 41 were stage IA (74.5%). Four patients exhibited FIGO stage II (7.3%) and the remaining four were stage III (7.3%).

Following the surgical procedure, 54 patients (98.2%) had no residual tumors, whereas one patient (1.8%) exhibited a macroscopic residual tumor of ≤2 cm and peritoneal carcinomatosis. Following surgery, two patients (3.6%) were treated with adjuvant platinum-based combination chemotherapy (175 mg/m^2^ paclitaxel; area under the curve 5 carboplatin dosage) every three weeks for six cycles for stage IIC and IIIC tumors, respectively, whereas the remaining 53 patients (96.4%) received no treatment. The two patients who received adjuvant chemotherapy achieved a complete response to the treatment.

A second surgical procedure was performed in five patients (9.1%) at the University of Bari following a first surgery performed at a peripheral hospital. All the anatomical sites that were removed and all the peritoneal cytology that was performed for these five patients were negative for the presence of disease.

### Outcome data

The median disease-free survival time and the five-year survival rate of the patient population were 42 months (range, 16–84 months) and 97%, respectively. Survival rate in the patients who underwent fertility-sparing surgery did not differ from that of the patients who had a complete surgical staging (P=0.08). Also, no differences were observed in survival rate after the results were stratified for stage (stage I-II vs. stage III; P=0.74), histological type (serous vs. mucinous, endometrioid and mixed tumor; P=0.15), tumor size (>10 vs. ≤10 cm; P=0.39), surgical approach (laparotomy vs. laparoscopy; P=0.56) and elevation of CA-125 at diagnosis (positive vs. negative marker; P=0.55).

Six patients (10.9%) relapsed and of these patients, four had received conservative surgery and the other two had received complete surgical staging at the time of the diagnosis. However, in all cases, a laparotomic approach was used. All patients underwent a surgical procedure for the relapse. Due to the presence of invasive implants in two of the six patients with recurrence at the time of diagnosis, adjuvant chemotherapy was performed after the first surgery. However, all six patients exhibited disease-free survival, with a median survival time of 39 months.

The fertility status was obtained for the 16 patients who underwent a fertility-sparing surgery. Four patients became pregnant and the remaining patients did not desire fertility at the time of their last follow up. One of these pregnancies was due to *in vitro* fertilization resulting in a successful pregnancy, whereas the remaining three were spontaneous.

## Discussion

BOTs represent an independent group of ovarian neoplasms with atypical epithelial proliferation and without stromal invasion. At present, no prospective randomized trials investigating the clinical management of BOTs have been conducted. However, BOTs have an excellent prognosis, with early-stage disease (stage I-II) exhibiting a five-year overall survival rate of almost 100% and with more advanced disease (stage III-IV) demonstrating a rate of 86–92% (9).

The current study has presented similar results to those described in the literature, such as an excellent prognosis with a five-year survival rate of 97%. Six patients developed recurrence. Due to the presence of invasive implants, two patients with recurrence were treated with surgery and chemotherapy following diagnosis. All patients were treated with surgery for the recurrence and at the present time, they exhibit no evidence of the disease.

Surgery epitomizes the ideal treatment. The role of surgery in the management of BOTs includes complete abdominal hysterectomy, omentectomy, peritoneal washings, bilateral salpingo-oophorectomy and various biopsies, including pelvic and para-aortic lymph node sampling. The rationale of this approach is that numerous apparent BOTs on frozen sections are found to be clear malignancies in permanent sections. Since BOTs often occur in patients during their childbearing years, numerous studies have proposed fertility-sparing surgery for those patients who desire to retain their reproductive function (10,11). Although numerous patients do undergo conservative surgery following the diagnosis, there have been limited studies on the safety and efficacy of fertility-sparing procedures (12,13). The rate of recurrence of BOTs following fertility-sparing surgery is reported to range between 0 and 30% (14), and certain studies have found these figures to be higher when compared with those patients whose surgery included hysterectomy and bilateral salpingo-oophorectomy. For example, Zanetta *et al* found that the recurrence rate in patients treated with fertility-sparing surgery was 19% compared with 5% in those who underwent more extensive surgery (14). Nevertheless, several studies have suggested that the overall disease-specific survival rates between the two surgical approaches are not different (11,12). Thus, it appears that young patients who desire future fertility can be safely treated with fertility-sparing surgery without compromising their overall survival.

In the present study, similar results to those of the aforementioned studies were observed, revealing no difference in survival rate between the two surgical approaches. All of those who relapsed were treated with conservative surgery at the time of diagnosis. However, despite these relapses, the patient prognosis was excellent, as all patients experienced disease-free survival. Therefore, based on the analysis of a small number of patients, re-operation solely for staging purposes would not have affected the outcome, even if conservative surgery is considered to be tenable in early-stage BOTs (15) and leads to a significantly increased risk of recurrence in advanced stages (15–17). In those who desire pregnancy and exhibit disease of an early FIGO stage, fertility-sparing surgery should be recommended, whereas for advanced-stage disease, complete surgical staging is strictly recommended.

Diagnostic laparoscopy has received considerable attention in the surgical management of patients with BOTs (18). No difference in survival was observed between the two surgical procedures in the present study.

According to the literature (19), serous-type tumors are frequently associated with elevated CA-125 levels rather than CA 19-9, which is associated more with mucinous-type tumors. Numerous studies have reported that invasive peritoneal implants are associated with a poor prognosis (20). It has been shown that serous tumors with a micropapillary pattern are a poor prognostic factor, as the tumors may frequently recur as an invasive carcinoma (17). These tumors confer a 10-year survival rate of ~60% in advanced-stage patients. In the present analysis, two out of six patients who relapsed possessed peritoneal implants at the time of the primary surgery and are therefore on a follow-up program. The present results revealed that, even in the presence of recurrent disease, the prognosis for patients with BOTs is excellent.

However, based on the data available in the literature, confirmed by the present results, the use of adjuvant chemotherapy for BOTs is a controversial subject. Currently, no study has proven adjuvant therapy to be beneficial, even in advanced-stage disease and with the presence of invasive implants (21). In the present study, only two patients underwent chemotherapy due to invasive implants and at the present time, they are experiencing disease-free survival.

In conclusion, BOTs have an excellent prognosis. Conservative surgery should be considered for patients of reproductive age who desire preservation of fertility. However, long-term follow-up is highly recommended for patients with these tumors.
